# A Novel *Bifidobacterium/Klebsiella* Ratio in Characterization Analysis of the Gut and Bile Microbiota of CCA Patients

**DOI:** 10.1007/s00248-023-02318-3

**Published:** 2023-11-30

**Authors:** Ningning Zhang, Wenwen Zhu, Shuwen Zhang, Tian Liu, Lan Gong, Zeyu Wang, Wei Zhang, Yunlong Cui, Qiang Wu, Jingtong Li, Hao Yu, Emad M. El-Omar, Jihui Hao, Wei Lu

**Affiliations:** 1 Department of Hepatobiliary Oncology, Tianjin Medical University Cancer Institute and Hospital, Liver Cancer Center, National Clinical Research Center for Cancer, Key Laboratory of Cancer Prevention and Therapy, Tianjin’s Clinical Research Center for Cancer, Tianjin Medical University, Tianjin, China; 2 Department of Medicine, Research and Education Centre Building, University of New South Wales, Level 2, 4-10 South Street, Sydney, Australia; 3 Microbiome Research Centre (MRC), St George and Sutherland Clinical School, University of New South Wales, Sydney, Australia; 4 Department of Hepatobiliary Surgery, Tianjin Medical University Cancer Institute and Hospital, National Clinical Research Center for Cancer, Key Laboratory of Cancer Prevention and Therapy, Tianjin’s Clinical Research Center for Cancer, Tianjin Medical University, Tianjin, China; 5 Department of Pancreatic Cancer, Tianjin Medical University Cancer Institute and Hospital, National Clinical Research Center for Cancer, Key Laboratory of Cancer Prevention and Therapy, Tianjin’s Clinical Research Center for Cancer, Tianjin Medical University, Tianjin, China

**Keywords:** Cholangiocarcinoma, Gut microbiome, Bile microbiome, Microbiomarkers, B/K ratio

## Abstract

Cholangiocarcinoma (CCA) is a serious health problem worldwide. The gut and bile microbiota have not been clearly characterized in patients with CCA, and better noninvasive diagnostic approaches for CCA need to be established. The aim of this study was to investigate the characteristics of the gut and bile microbiota in CCA patients. Forty-two CCA patients and 16 healthy normal controls (HNCs) were enrolled. DNA was extracted from fecal and bile samples and subjected to 16S rRNA gene analysis. We found that there were significant differences in the species diversity, structure, and composition of the microbial communities between the CCA group and the HNC grouAt the phylum level, compared with that in the HNC group, the relative abundance of Firmicutes and Actinobacteriota was significantly decreased in the CCA group, whereas Proteobacteria and Bacteroidota were significantly enriched. The Firmicutes/Bacteroidota (F/B) ratio significantly decreased in the CCA group compared to the HNC grouThe relative abundance of *Klebsiella* in the CCA group was significantly higher than that in the HNC group, while the relative abundance of *Bifidobacterium* was significantly decreased. The *Bifidobacterium/Klebsiella* (B/K) ratio was established as a novel biomarker and was found to be significantly decreased in the CCA group compared with the HNC grouOur findings provide evidence supporting the use of *Klebsiella* and *Bifidobacterium* as noninvasive intestinal microbiomarkers for improving the diagnosis of CCA.

**Supplementary Information:**

The online version contains supplementary material available at 10.1007/s00248-023-02318-3.

## Introduction

Cholangiocarcinoma (CCA) is a rare cancer that may arise at any site in the biliary tree. The incidence of CCA is 0.3–6 per 100,000 inhabitants per year and the mortality is 1–6 per 100,000 inhabitants per year, globally. Although considered a rare cancer, the incidence of CCA is increasing [1]. CCA is classified into the following subtypes according to the anatomical location: intrahepatic CCA, perihilar CCA, or distal CCA [2]. The cause for a majority of CCAs is unknown, but there are several risk factors that may predispose individuals to CCA development (age, obesity, diabetes, inflammatory liver diseases, hepatolithiasis, cirrhosis, infectious agents, and congenital disorders) [3]. The diagnosis of CCA is based on a combination of clinical, radiological, biochemical, and histological approaches. Currently, highly specific and sensitive biomarkers to assist in the diagnosis of this disease are lacking [4]. Only in a small proportion of patients is CCA detected in early stages, and surgical resection is a potentially curative treatment, significantly increasing the overall survival of patients. Therefore, noninvasive methods that use highly specific and sensitive biomarkers for diagnosing CCA are urgently needed.

Inflammation is one of the hallmarks of cancer, and the pro-inflammatory effects of markers of systemic inflammatory response (SIR) that often reflect cancer immune status, such as plasma C-reactive protein (CRP), platelet to lymphocyte ratio (PLR), neutrophil to lymphocyte ratio (NLR), and lymphocyte to monocyte ratio (LMR), have been shown to play an important role in the development and progression of cancer. SIR is also a potential prognostic and/or predictive factor for hepatocellular carcinoma, colorectal cancer, and other malignancies [5]. The albumin-to-alkaline-phosphatase ratio (AAPR) is an index based on serum biomarkers, which may not only reflect the liver functional reserve but also be associated with inflammation and cancer cell proliferation [6]. High NLR, PLR, and CRP and low LMR and AARP were associated with advanced clinicopathological features and poor prognosis [5–7], but few studies had been done in CCA patients.

More than 100 trillion microorganisms present in the gut form a complex microbial community whose dynamic balance maintains normal gastrointestinal homeostasis that has a major impact on human health [8]. The gut microbiome is increasingly being recognized for its influence on health and disease, including cancer. For example, certain bacteria and viruses have been implicated in cellular dysplasia and carcinogenesis [9]. The gut-liver axis is closely related to the function of the gut and hepatobiliary systems. The hepatobiliary system is closely related to the gut in terms of anatomical position and physiological function. Dysbiosis of the gut microbiome and increased permeability of the intestinal wall are closely related to hepatobiliary disease through immune responses [10]. Biliary obstruction caused by CCA leads to cholestasis and thus reduced biliary secretion, which provides a medium for bacteria. Low immunity in CCA patients and decreased biliary secretion can promote the overgrowth of the gut microbiome, resulting in the increasing abundance of pathogenic bacteria and changes in microbiome abundance [11, 12]. Because of its anatomical location, the liver is affected by the composition of gut bacteria, which means that gut bacterial products can spread to the liver through the portal vein [13]. A growing number of studies have found that bile also contains a microbiome that may influence bile metabolism [14, 15].

Some studies have examined the tumor and gut microbiomes, and some have established noninvasive diagnostic models based on the gut microbiome, achieving good performance [16]. However, few studies have examined the gut microbiome of CCA. Moreover, the changes in the bile microbiome of CCA patients have rarely been studied. Therefore, in this clinical study, we wanted to further study the relationship between the gut microbiota and CCA to gain a better understanding of the underlying interactions. In order to understand the influence of the fecal microbiome on CCA patients, this study intends to further use 16S rRNA amplicon sequencing and microbiome analysis to study the differences between the fecal microbiome of CCA patients and normal specimens in classification, clarify the intestinal microbiome characteristics of CCA, and further explore the characteristics of bile microbiome.

## Materials and Methods

### Patient Clinical and Laboratory Data Collection

A total of 42 patients who were diagnosed with CCA at the Department of Liver Cancer Center, Tianjin Medical University Cancer Institute and Hospital, between January 2021 and December 2021, were enrolled in this study. The initial diagnosis of CCA was determined by pathology or imaging methods (CT/MRI). Bile and fecal samples of patients with CCA were collected before treatment was initiated. The exclusion criteria of patients with CCA were as follows: (1) CCA concomitant with other malignant tumors; (2) patients who had received antitumor therapy; (3) CCA combined with chronic diseases such as gastrointestinal diseases; (4) use of antibiotics, proton pump inhibitors, or probiotic products 1 month before sampling; and (5) unavailability of clinical parameters. Sixteen healthy normal controls (HNCs) were enrolled, and the exclusion criteria were listed as follows: (1) ≤ 18 years old or ≥ 80 years old; (2) use of antibiotics, proton pump inhibitors, or probiotic products one month before sampling; (3) diagnosed with malignancies or with gastrointestinal disorders. This study was approved by the institutional review board of the medical center and written informed consent was obtained from all participants.

To date, one of the most studied and most used tumor markers in cholangiocarcinoma is carbohydrate antigen 19-9 (CA 19-9) [17]. CCA patients with CA19-9 positive (CCP group) were divided into the following two groups according to the cut-off value of 10 times the upper limit of normal serum CA19-9 levels (270 U/mL); we have defined patients within 10 times CA19-9 levels (27 < CA19-9 value < 270) as CA19-9 positive low group (CPL group) and others (CA19-9 value≥270) as CA199 positive high group (CPH group), and the third group was those with CA19-9 negative (CCN group).

### DNA Isolation from Fecal Samples and 16S rRNA Amplicon Sequencing

Fecal and bile samples were collected from each participant and immediately stored in a −80 °C freezer for subsequent analysis. Bile samples were collected from the common bile duct during routine endoscopic retrograde cholangiopancreatography (ERCP) [18, 19]. The collected specimens were centrifuged at 7500 × g and 4 °C for 10 min. The CTAB method was employed for the extraction of total genomic DNA from the bile [20, 21]. A fecal DNA Extraction Kit (Tiangen, China) was used to extract total genomic DNA from the samples. The extracted DNA was amplified by specific primers targeting the V3 to V4 region (250-bp paired-end reads) of the 16S rRNA gene. The primer sequences were the following: 341F: 5′-CCTAYGGGRBGCASCAG-3′, 806R:5′-GGACTACNNGGGTATCTAAT-3′ [22]. The PCR process was as follows: 98 °C for 1 min, followed by 30 cycles (of 95 °C for 10 s, 55 °C for 30 s, 72 °C for 30 s, and 72 °C for 5 min). After the PCR process, products were detected by electrophoresis on a 2% agarose gel. Then, a Qiagen Gel Extraction Kit (Qiagen, Germany) was used to purify the PCR product mixture. Sequencing libraries were produced by a TruSeq® DNA PCR-Free Sample Preparation Kit (Illumina, USA) following the manufacturer’s recommendations, and index codes were added. Finally, the library was sequenced on an Illumina NovaSeq platform, and 250-bp paired-end reads were generated.

### Bioinformatics and Statistical Analyses

With reference to the tags, quality control process of Qiime (V1.9.1), high-quality, clean, and effective tags are obtained by strict filtering and processing of raw tags. Uparse software (Uparse v7.0.1001) was used for the sequence analysis. Sequences with more than 97% similarity were defined as belonging to the same OTUs. The Silva database (http://www.arb-silva.de/) based on the Mothur algorithm was applied to annotate the taxonomic information of each representative sequence. Alpha diversity indices were applied to analyze the complexity of species diversity for each sample, including the Chao1 and Shannon indices. Alpha diversity indices [22, 23] were calculated with QIIME (Version 1.9.1) and visualized by R software. Beta diversity analysis was used to evaluate differences in species complexity [22, 23]. Beta diversity analysis was performed by QIIME software (version 1.9.1). Principal coordinate analysis (PCoA) was performed to obtain principal coordinates and visualize complex, multidimensional data. PCoA results were visualized by the WGCNA package, stat packages, and ggplot2 package in R software. The Shapiro-Wilk test was used to determine the normality of the data. To determine the enrichment in the assigned taxonomic and functional profiles, LEfSe analysis was performed. Taxonomic levels with LEfSe values higher than 4 at a *P* value < 0.05 were considered statistically significant. The differential microbial features of groups were identified using statistical analysis of metagenomic profiles (STAMP) [23]. General statistical analysis was performed in GraphPad Prism (GraphPad Software, Inc.). One-way ANOVA with Tukey’s multiple comparisons test was used to determine differences between groups. Spearman’s rank correlation analysis was performed in R (v3.6.1) to analyze the relationships between the microbiome and disease status. *P* values < 0.05 were considered to indicate statistical significance. Operating characteristic curves (receiving operational curve, ROC) were constructed, and then the area under the curve (AUC) was calculated to evaluate the discriminatory ability of gut microbiome signature in different groups.

## Results

### Alpha and Beta Diversity of the CCA Sample Microbiomes

**Fig. 1:**
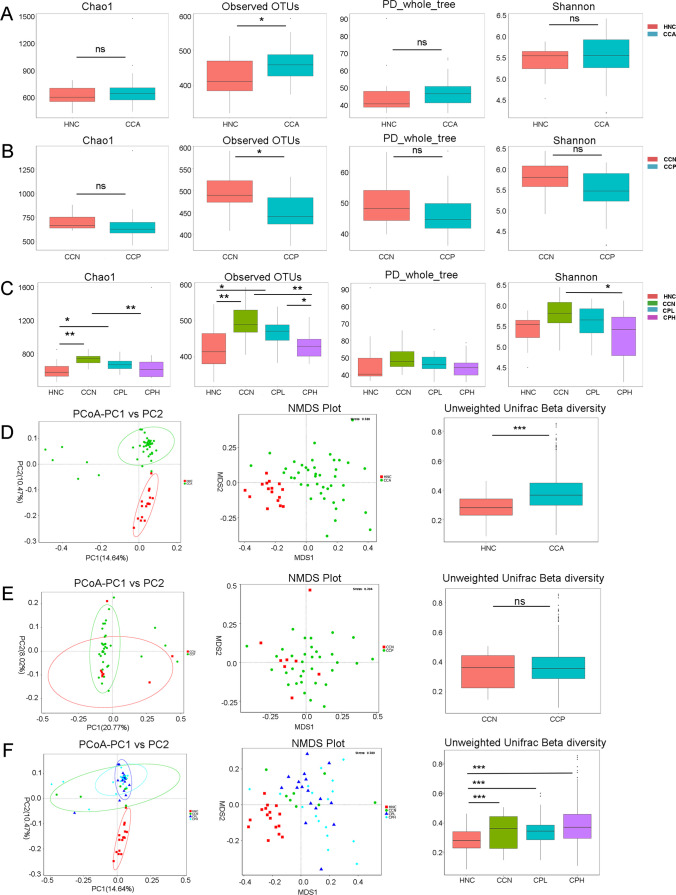
Alpha diversity and beta diversity. Comparison of the alpha diversity of the gut microbiota between CCA patients and the HNC group (**A**), between the CCN and CCP groups (**B**), and among the HNC, CCN, CPL, and CPH groups (**C**) using the Shannon and PD_whole_tree, observed OTU and Chao1 indices to evaluate bacterial diversity, richness, and abundance, respectively. Comparison of the beta diversity of the gut microbiota between CCA patients and the HNC group (**D**), between the CCN and CCP groups (**E**), and among the HNC, CCN, CPL, and CPH groups (**F**) using unweighted PCoA, NMDS, and unweighted Unifrac beta diversity. Each point represents an individual sample. Differences in microbiota composition between the groups are displayed as a box plot according to the Wilcoxon rank sum test; *P < 0.05, **P < 0.01, ***P < 0.001

Comparison of α-diversity metrics showed that CCA patients had a significantly higher bacterial richness (observed OTUs) representing the number of species relative to the healthy normal control (HNC) group (Fig. 1A; *P* < 0.05), but there was no significant difference in bacterial diversity (Shannon diversity and PD_whole_tree) and abundance (Chao1) (Fig. 1A). CCP patients had significantly lower bacterial richness (observed OTUs) representing the number of individuals of each species (*P* < 0.05) than the CCN group, but there was no significant difference in bacterial diversity (Shannon diversity and PD_whole_tree) or abundance (Chao1) (Fig. 1B). Interestingly, we found that CCN patients had significantly higher bacterial richness (observed OTUs) (*P* < 0.05) and abundance (Chao1) (*P* < 0.05) than the HNC group, and with the increase in the CA19-9 level, the observed OTUs and Chao1 gradually decreased (Fig. 1C).

Unweighted PCoA, nonmetric multidimensional scaling (NMDS), and beta diversity box plots were used to assess beta diversity. The beta diversity of the CCA patients and HNC group exhibited a significant difference (Fig. 1D; *P* < 0.001), but there was no significant difference between the CCN and CCP groups (Fig. 1E). Subgroup analysis demonstrated that there was a significant difference between the CCN, CPL, and CPH groups and the HNC group (Fig. 1F; *P* < 0.05). In summary, the composition of the gut microbiota is altered in CCA patients and is highly diverse.

### Differences in Gut Microbiome Composition Between the HNC Group and CCA Patients at the Phylum Level

**Fig. 2:**
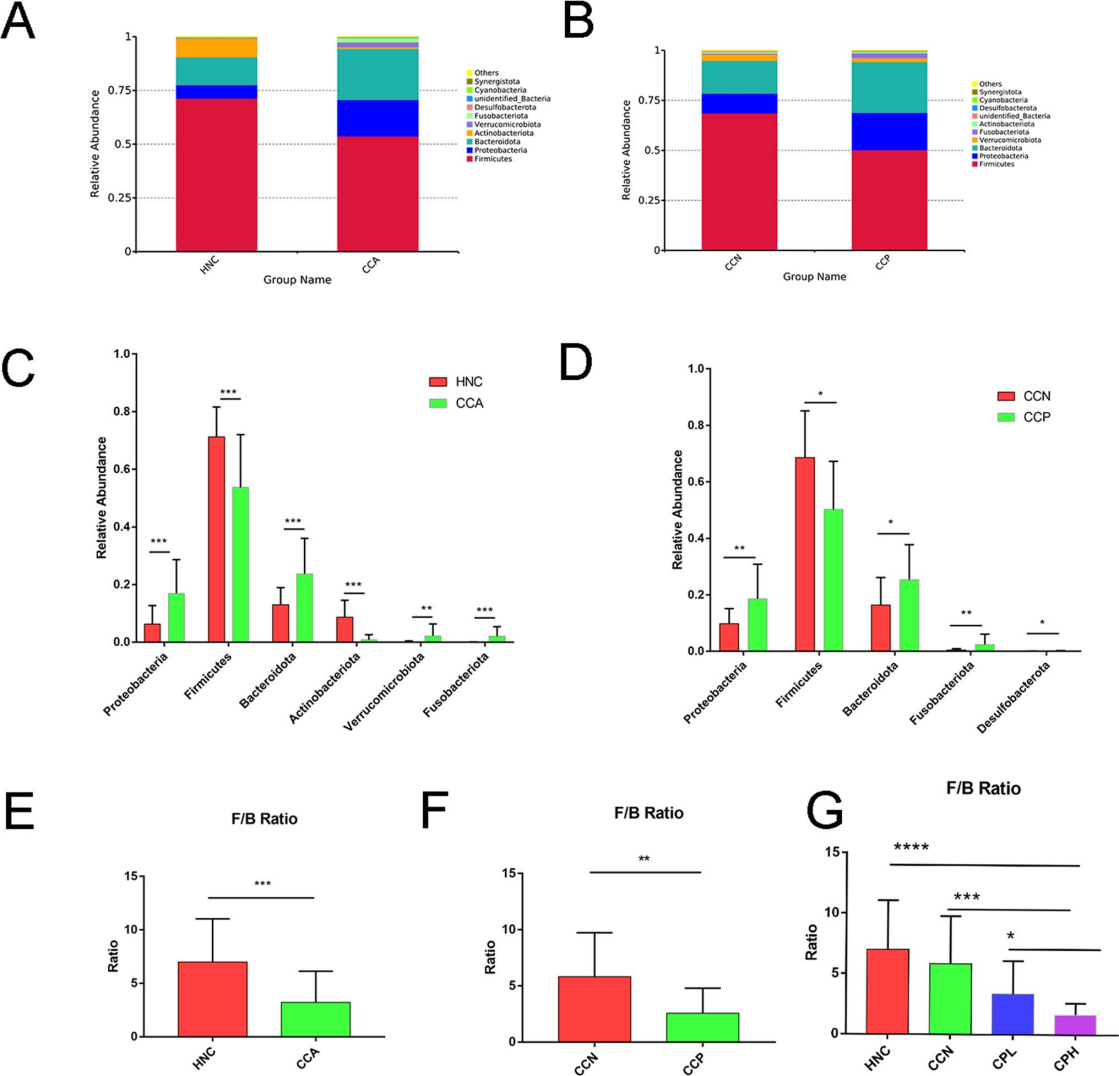
The compositional difference between the HNC and CCA groups at the phylum level. Stacked bar plot of the mean proportions of taxonomic composition in the HNC and CCA groups (**A**) and in the CCN and CCP groups (**B**) at the phylum level. Box plot showing the relative abundances of the top 6 (or 5) differentially abundant taxa identified by a stacked bar plot in the HNC and CCA groups (**C**) and in the CCN and CCP groups (**D**). Firmicutes/Bacteroidota ratio in the HNC and CCA groups (**E**), CCN and CCP groups (**F**), and HNC, CCN, CPL, and CPH groups (**G**). *P < 0.05, **P < 0.01, ***P < 0.001, ****P < 0.0001

Firmicutes, Actinobacteriota, Bacteroidota, Proteobacteria, and Verrucomicrobia were the five dominant phyla in the HNC and CCA groups. According to the bar plots of taxon abundance at the phylum level, the phyla Firmicutes and Bacteroidota were the most abundant phyla in the two groups (Fig. 2A). Similar results were also observed for the CCN and CCP groups (Fig. 2B). The CCA group had a lower average abundance of Firmicutes and Actinobacteriota than the HNC group, and the average abundance of Bacteroidota, Proteobacteria, and Verrucomicrobia in the CCA group was higher than that in the HNC group (Fig. 2C). However, the CCN group had a higher average abundance of Firmicutes than the CCP group, and the average abundance of Actinobacteriota, Bacteroidota, Proteobacteria, and Verrucomicrobia in the CCN group was lower than that in the CCP group (Fig. 2D).

Firmicutes and Bacteroidota are the most common bacterial phyla constituting the human microbiome. Therefore, the Firmicutes/Bacteroidota (F/B) ratio is used as a representative index to compare different microbial communities. Many studies have supported the idea that a high F/B ratio signifies healthy conditions. Additionally, a corresponding decrease in the F/B ratio was observed in patients with CCA (Fig. 2E), which could be an important marker for intestinal dysbiosis. There was a significant difference in the F/B ratio between the CCN and CCP groups (Fig. 2F). The HNC group had a higher F/B ratio than the CCN group, though the difference was not significant, and had a higher F/B ratio than the CPL and CPH groups, and the differences were statistically significant. Although the F/B ratio can be used to distinguish healthy and disease conditions, it cannot be used to effectively distinguish healthy and CA19-9-negative CCA patients (Fig. 2G). Detailed information of Firmicutes and Bacteroidota is listed in Table S1. Therefore, it is necessary to identify a new index to better distinguish healthy and disease conditions.

### HNC and CCA Patients Show Differing Bacterial Communities, Especially in Terms of the Abundance of *Klebsiella* and *Bifidobacterium*

**Fig. 3:**
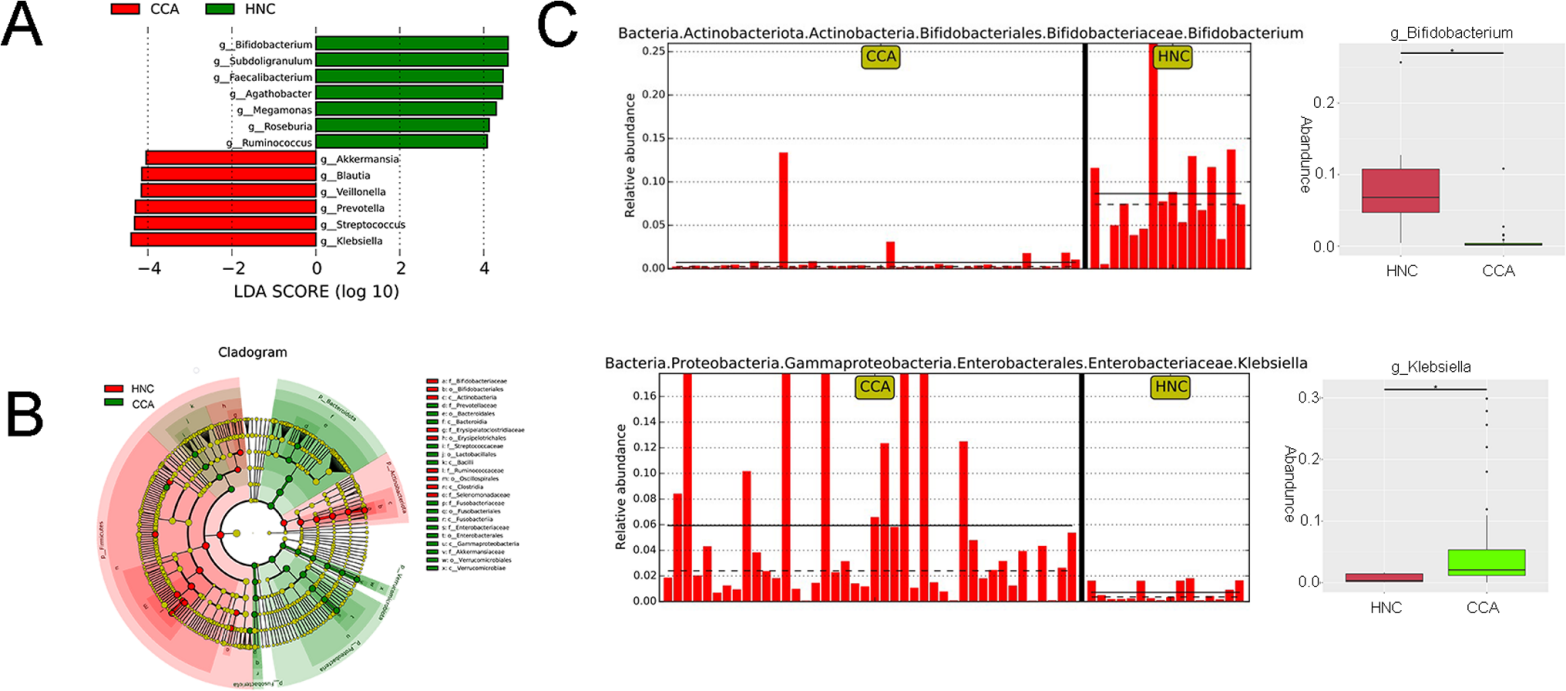
LEfSe analysis of the gut microbiota. **A** LEfSe analysis showed significant differences in bacterial abundances between the HNC and CCA groups. **B** Cladogram of different taxa between the HNC and CCA groups. *P < 0.05

To comprehensively consider the biological consistency and effect size, taxonomic analysis using the LEfSe was carried out. The different phylogenetic relationships with the criterion LDA ≥ 4 between every pair of groups are shown. LEfSe showed that there were 13 taxa with different distributions at the genus level among the HNC and CCA groups (Fig. 3A). The predominant bacteria and different taxa among the groups are indicated in the phylogenetic tree, and the results revealed the different enriched taxa between the two groups in the HNC and CCA groups and the existence of dysbiosis in the CCA group (Fig. 3B). The genus *Klebsiella* was found to be the most significantly enriched in the CCA group (*P* < 0.05, LDA score > 4; Figure S1), while the genus *Bifidobacterium* was found to be the most significantly enriched in the HNC group (*P* < 0.05, LDA score > 4; Figure S2). So, we selected the two taxa (*Bifidobacterium* and *Klebsiella*) from 13 showing significant differential abundance between groups for further study to explore their potential biological significance. Briefly, significant differences in the gut microbiota indeed existed between the HNC group and CCA patients.

The genus *Klebsiella* was significantly enriched in the CCA group, while the genus *Bifidobacterium* was significantly enriched in the HNC group, as determined by LEfSe analysis. To further identify the taxa that contributed to the observed differences between the intestinal microbiomes of the CCA and HNC groups, we also analyzed the differentially abundant species. The results showed that the species *Klebsiella variicola* was significantly enriched in the CCA group, while the species *Bifidobacterium adolescentis* and *Bifidobacterium longum* were significantly enriched in the HNC group (Figure S3 and S4). The relative abundance of the genus *Klebsiella* increased with increasing CA19-9 levels (Figure S5 and S6). The relative abundance of *K. variicola* was significantly higher in the CCN group than in the HNC group, while the abundance of *Bifidobacterium adolescentis* was significantly higher in the HNC group than in the CCN group (Figure S4). Therefore, the genera *Klebsiella* and *Bifidobacterium* may play an important role in the development of CCA.

**Fig. 4:**
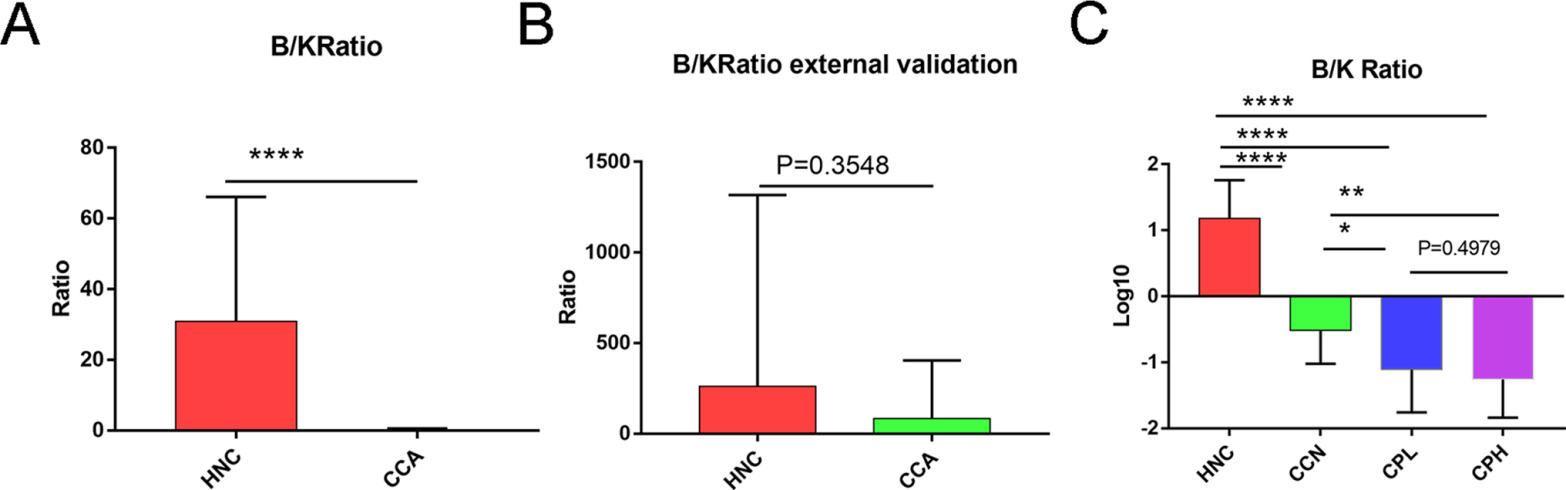
*Bifidobacterium/Klebsiella* (B/K) ratio in different groups. **A** *Bifidobacterium/Klebsiella* ratio in the HNC and CCA groups. **B** *Bifidobacterium/Klebsiella* ratio in the HNC and CCA groups for external validation. **C** *Bifidobacterium/Klebsiella* ratio in the HNC, CCN, CPL, and CPH groups. *P < 0.05, **P < 0.01, ***P < 0.001, ****P < 0.0001

To further study the role of *Klebsiella* and *Bifidobacterium* in CCA patients, we established the *Bifidobacterium/Klebsiella* (B/K) ratio as an index to further explore the characteristics of the HNC and CCA groups. The study showed that the B/K ratio was significantly reduced in CCA patients compared to the HNC group (Fig. 4A), and external data validation yielded similar results (Fig. 4B). Detailed information on *Bifidobacterium* and *Klebsiella* is listed in Table S1. External validation data (Supplementary Table 2) was proceeded with the microbiome data from the published research article of Gang Chen [22]. As the CA19-9 level increased, the B/K ratio decreased gradually. Importantly, the B/K ratio can distinguish HNC patients from CCN patients very well, compensating for the deficiency of the F/B ratio.

### Clinical Characteristics of CCA Patients in the Study and Associations Among Inflammatory Response Markers, the CONUT Score, and the Tumor Marker CA19-9 in CCA

**Fig. 5:**
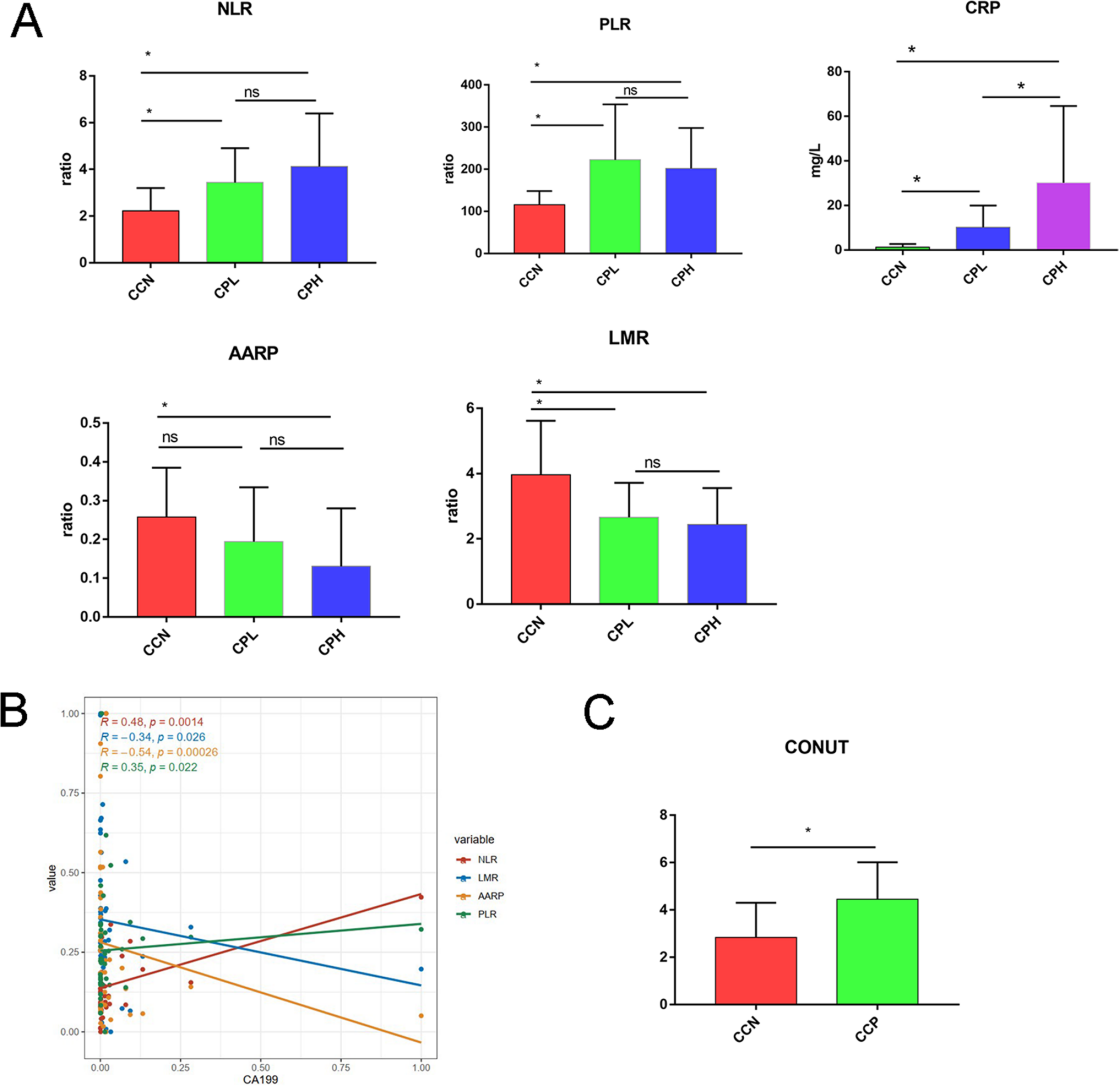
Clinical characteristics of CCA patients. **A** Association between CA19-9 and other clinical characteristics (NLR, PLR, AARP, LMR, CRP). **B** Correlations among the NLR, PLR, LMR, AARP, PLR, and CA19-9 in the CCA grou**C** CONUT scores of the CCN and CCP groups. *P < 0.05, ***P < 0.001

Clinical information for 42 CCA patients, including 8 CCN patients and 34 CCP patients, was collected and analyzed. The clinicopathological information is presented in Supplementary Table 3 and Supplementary Table 4. CA19-9 is a commonly used marker for the diagnosis of CCA. Recently, numerous studies have indicated that hematological markers, for instance, the CRP, NLR, PLR, LMR, and albumin-to-alkaline phosphatase ratio (AAPR), may serve as inflammatory markers. The PLR, NLR, LMR, and AAPR have been shown to serve as predictive factors for determining CCA prognosis and treatment outcomes. We intended to further investigate the relationship between inflammatory response markers and the tumor marker CA19-9 in CCA. The CCN group showed lower NLR (*P* < 0.05), PLR, and CRP levels and higher LMR and AAPR levels than the CPL and CPH groups (Fig. 5A, Supplemental Table 1). Furthermore, Spearman analysis was used to investigate the relationship between inflammatory response markers and CA 19-9. CA19-9 was positively correlated with the NLR and PLR but negatively correlated with the LMR and AAPR in CCA patients (all *P* < 0.05) (Fig. 5B).

The controlling nutritional status (CONUT) score system, a system for scoring immune nutritional status that was developed in 2005, has garnered the attention of researchers; it includes the measurement of serum albumin and total cholesterol levels as well as peripheral blood lymphocyte levels (Supplemental Table 2). A growing body of evidence has suggested that patients with high CONUT scores generally have poor nutritional and protumor immunity statuses, potentially leading to tumor invasion and metastasis. Our study showed that the CCP group had a higher CONUT score (*P* < 0.05) than the CCN group (Fig. 5C). Spearman analysis was used to investigate the relationship between inflammatory response markers, CA 19-9, and B/K ratio (Supplementary Figure 4).

### Differences in the Gut and Bile Microbiome Compositions in CCA Patients

**Fig. 6:**
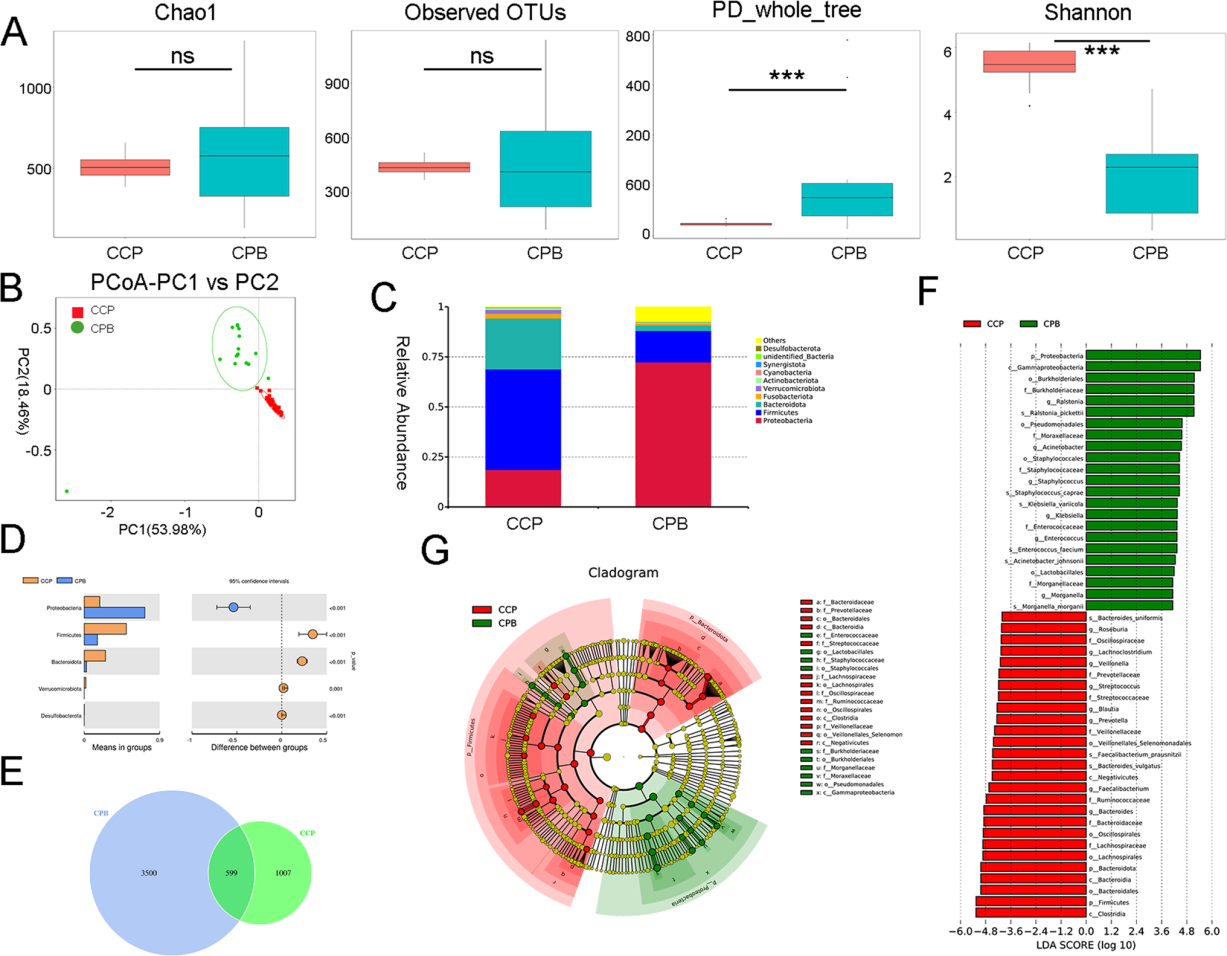
Differences in the gut and bile microbiome compositions in CCA patients. **A** Comparison of alpha diversity of the gut microbiota between CCP patients and the CPB grou**B** Comparison of beta diversity assessed by weighted PCoA of the gut microbiota between CCP patients and the CPB grou**C** Stacked bar plot of mean proportions of taxonomic abundances in CCP patients and the CPB grou**D** Box plot showing the relative abundances of the top 5 differentially abundant taxa identified by a stacked bar plot. **E** Venn diagram exhibiting shared and unique OTUs between the CCP and CPB groups. **F** LEfSe analysis showed significant differences in bacterial abundance between the CCP and CPB groups. **G** Cladogram of different taxa between the CCP and CPB groups. ***P < 0.001

We also performed microbiological analysis of bile and stool samples in the CCP group to further explore the potential microbiological characteristics of CA19-9-positive patients. Comparison of α-diversity metrics showed there was a significant difference in bacterial diversity (Shannon diversity and PD_whole_tree) (*P* < 0.001) between the bile of the CCP (CPB) group and the CCP group, but there was no significant difference in bacterial richness (observed OTUs) and abundance (Chao1) (Fig. 6A). This indicates that the CCP group had relatively higher community diversity and relatively uniform species distribution than the CPB group and that species differences existed between the two groups. Weighted PCoA was used to assess beta diversity. The composition of the gut microbiota was altered in the CCP and CPB groups and differed between the two groups (Fig. 6B). According to the bar plots of taxonomic abundances at the phylum level, the phyla Bacteroidota and Firmicutes were the most abundant phyla in the CCP group, while the phyla Firmicutes and Proteobacteria were the most abundant phyla in the CPB group (Fig. 6C). The CPB group had a lower average abundance of Firmicutes, Bacteroidota, Actinobacteriota, and Verrucomicrobia than the CCP group, and the average abundance of Proteobacteria in the CPB group was higher than that in the CCP group (Fig. 6D). Through Venn diagram analysis, we found that the CPB group had significantly more OTUs than the CCP group (Fig. 6E). LEfSe showed that there were taxa with different distributions between the CCP and CPB groups (Fig. 6F). The taxonomic analysis using LEfSe with the criterion LDA ≥ 4 is also shown in the cladogram (Fig. 6G).

### Gut Microbiota and Serum Tumor Marker-Based Classification Model Construction and Validation for the Prediction of CCA

**Fig. 7:**
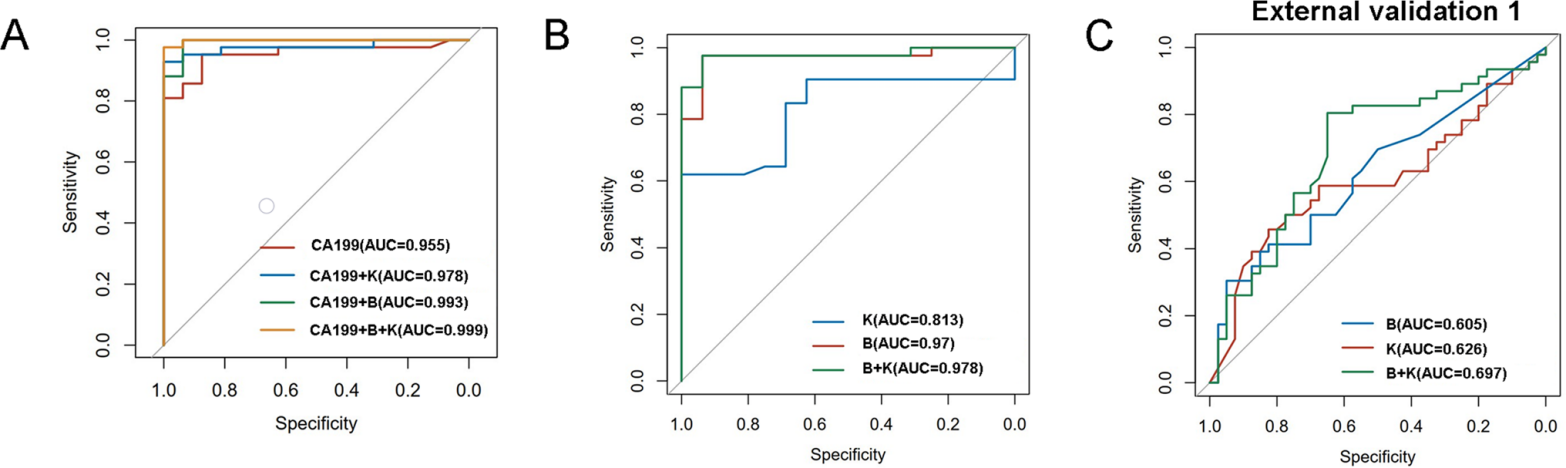
Gut microbiota and serum tumor marker-based classification model construction and validation for the prediction of CCA. **A** ROC curve analysis to evaluate the classification ability of the serum tumor marker CA19-9 combined with the gut microbiome signature in predicting different groups. **B** ROC curve analysis in the HNC and CCA cohorts combined to evaluate the classification ability of the gut microbiome signature in predicting different groups. **C** ROC curve analysis in the external validation HNC and CCA cohorts combined to evaluate the classification ability of the gut microbiome signature in predicting different groups. The combination of the gut microbiome signature and CA19-9 was performed using the logistic regression method

To construct the classification model to distinguish between the HNC group and CCA patients, an ROC curve was used to characterize biomarkers based on the serum tumor markers CA19-9, *Bifidobacterium*, and *Klebsiella*. Our above study, using LefSe analysis, showed that *Klebsiella* had the highest relative abundance in the CCA group while *Bifidobacterium* had the highest relative abundance in the HNC group at the genus level, showing a significant difference in the relative abundances of *Bifidobacterium* and *Klebsiella* in the HNC and CCA groups. We established a training cohort of 16 HNC and 42 CCA patients and a validation cohort of 40 HNC and 46 CCA patients from the published research article of Gang Chen [22]. The classification accuracy of this model in the prediction between the HNC group and CCA patients was tested using ROC curves. We evaluated the ability of tumor markers CA19-9 combined with *Bifidobacterium* and *Klebsiella* to distinguish between the HNC and CCA groups. CA19-9 had an area under the curve (AUC) of 0.955 in the classification of HNC and CCA. CA19-9 and *Klebsiella* combined using the logistic regression method also showed an AUC of 0.978. CA19-9 and *Bifidobacterium* combined showed an AUC of 0.993, while CA19-9, *Bifidobacterium*, and *Klebsiella* combined showed an AUC of 0.999, which was the highest classification score among the four predictive ability comparison cohorts of tumor markers CA19-9 combined with *Bifidobacterium* and *Klebsiella* in our study (Fig. 7A). To further compare the prediction ability of our classification model with that of classic clinical tumor markers for the HNC and CCA groups, ROC curve analysis in the HCC and CCA cohorts was combined to compare the classification ability of the gut microbiome signature for predicting different groups. The AUC comparison among the four predictive ability comparison cohorts of *Bifidobacterium* and *Klebsiella* without tumor markers CA19-9 confirmed the accuracy of classification among *Bifidobacterium*, *Klebsiella*, and *Bifidobacterium* combined with *Klebsiella* with values of 0.97, 0.813, and 0.978, respectively (Fig. 7B). *Bifidobacterium* combined with *Klebsiella* had higher accuracy of classification than each genus alone, which was verified by external study data (Fig. 7C), but the accuracy was lower than that of CA19-9, *Bifidobacterium*, and *Klebsiella* combined.

## Discussion

CCA is the second most common primary liver cancer, accounting for about 15%. Most patients with CCA are diagnosed at an advanced disease stage and have a poor prognosis due to a lack of obvious symptoms to promote early diagnosis [24]. The gastrointestinal system is one of the largest reservoirs of microorganisms in the human body, and it holds both commensal and pathogenic microbial species [25, 26]. To date, little is known about the composition of the biliary tract microbiota and its influence on the development of biliary diseases. Studies have shown that the gut microbiome can be used to explore potential biomarkers of CCA. In a case-control study, Zhang et al. [27] performed high-throughput sequencing of 16S rRNA from fecal samples of CCA patients, cholelithiasis patients, and healthy controls and proposed a predictive model based on intestinal microbiome characteristics to better diagnose CCA. Similarly, Deng et al. [22] conducted 16S rRNA sequencing from fecal samples of CCA patients, HCC patients, and healthy controls and established a prediction and screening model for HCC based on intestinal microbiome characteristics. Under healthy conditions, bacteria in the microbiome interact with the epithelial barrier and produce substances that affect local metabolism to maintain the structural integrity of the bowel epithelial barrier [28] and homeostasis, while the link between the development of CCA and gut microbiome has attracted more attention [12, 15, 29]. Most advanced CCA patients were admitted to the hospital with obstructive jaundice and underwent an ERCP examination to obtain tissue to confirm the diagnosis and resolve the jaundice. Therefore, more and more studies on the bile microbiome in CCA patients have been paid attention. This observed differential abundance in microbiome between CCP and CPB groups raises a possibility that the difference could be due to the nature of the tumor itself by its influence on the gut-liver axis and further alteration of the bidirectional communication between the gastrointestinal tract and the liver via the biliary tract, portal vein, and systemic circulation subsequently exposing biliary tract to the gastrointestinal microbiome [30]. Our study suggests that distinct microbiome signatures in bile are associated with CCA. Therefore, more and more studies on bile microbiome in CCA patients have been paid attention.

Probiotics such as *Bifidobacterium* and *Lactococcus* alter the composition of the gut flora by exerting antibacterial and anti-inflammatory effects at the mucosal surface and play antioxidant, anticancer, and immunomodulatory roles [31]. *Bifidobacterium*, the dominant member of gut probiotics microbiome, reduces the risk of intestinal infections by regulating immune responses and protecting against intestinal barrier dysfunction [32], which protects against cancer by downregulating EGFR and COX-2 [31, 33] and induces apoptosis in cancer cells via the activation of pro-apoptotic Bax and the inactivation of the anti-apoptotic Bcl-2 proteins [34]. Studies have also shown that the gut microbiome composition is an important factor determining the response to cancer therapeutics, such as anti-PD-1 treatment of melanoma patients [35]. *Bifidobacterium* was also previously shown to have a beneficial effect on ulcerative colitis in a clinical trial [36, 37]. The protective effects of *Bifidobacterium* against these diseases indicate the potential applicability of microbiome-based therapy. *Klebsiella* has been shown to be enriched in a variety of tumors (such as pancreatic cancer, colon cancer, and esophageal cancer) [38–40]. This genus is related to the lymphatic metastasis and prognosis of pancreatic cancer [38]. However, there are few studies on *Bifidobacterium* and *Klebsiella* in CCA patients, and the specific characteristics and effects of *Bifidobacterium* and *Klebsiella* in CCA patients remain unclear.

Our study showed that the genus *Klebsiella* was significantly enriched in the CCA groups, while the genus *Bifidobacterium* was significantly enriched in the HNC grouIt can be concluded that *Klebsiella* may promote or play an important role in the development of CCA, while *Bifidobacterium*, as a well-known genus of beneficial bacteria, protects the body from the development of CCA. In our study, it was found that *Bifidobacterium* and *Klebsiella* may have played an important role in the occurrence and development of CCA, so we established the ratio of *Bifidobacterium* and *Klebsiella* (B/K) as an index to further explore its role in CCA. We found that the B/K ratio in the CCA group was significantly lower than that in the HNC group and gradually decreased with increasing CA19-9 levels. Surprisingly, compared with the HNC group, CA199-negative CCA patients had a significantly lower B/K ratio, which could be used to distinguish their health status from that of CA199-negative CCA patients. The B/K ratio may be used as a noninvasive index for the early diagnosis of CCA. There is ample evidence showing that *Bifidobacterium* attenuates colitis and inhibits the development of CRC, including CAC [41]. In other words, *Bifidobacterium* has anti-inflammatory effects. *Klebsiella*, a common human respiratory pathogen, has proinflammatory effects. The B/K ratio explains the correlation between inflammation and CA19-9 at the microbial level. That is, the higher the CA19-9 level is, the lower the B/K value is, which better indicates the systemic inflammatory response of the body contributed by the microbiome.

We further conducted ROC curve analysis of CA19-9 with or without *Bifidobacterium* and *Klebsiella*, and the ROC curve showed that the *Bifidobacterium* combined with *Klebsiella* classification model had higher accuracy than each bacterial genus alone, which was verified by external study data, but the accuracy was lower than that of the CA19-9, *Bifidobacterium*, and *Klebsiella* combined classification model (AUC = 0.999) in our study. The CA19-9, *Bifidobacterium*, and *Klebsiella* classification model can improve the early diagnosis of CCA. We compared our gut microbiome-based model with serum tumor markers commonly used in clinical diagnosis and showed it to be superior. These results may indicate that this gut microbiome-based classification model is a potential noninvasive screening method for CCA.

In the present study, we performed a comprehensive intestinal microbiome analysis of the HNC group and CCA patients with different levels of the tumor marker CA19-9 (the CCN, CPL, and CPH groups). Firmicutes, Actinobacteriota, Bacteroidota, Proteobacteria, and Verrucomicrobia were the five dominant phyla in the HNC and CCA groups. Among them, the two most dominant phylum in the human intestine are Firmicutes and Bacteroidota, accounting for more than 98%, while the proportion of others only accounts for about 1% [42]. The major microbiome component of the HNC group and the CCA group was found to be Firmicutes (with a percentage of 50–75%), followed by Bacteroidota. Our research indicates that the CCA group had a lower average abundance of Firmicutes and a higher abundance of Bacteroidota than the HNC group, which is consistent with previous studies [22, 27]. The declined Firmicutes/Bacteroidota abundance ratio was seen as an indicator of microbial imbalance [43]. The F/B ratio has been reported as a potential marker of pathophysiologic conditions [44, 45]. The fecal F/B ratio may influence host metabolism and inflammation since it might play a potential anti-inflammatory role [46], and long-term chronic inflammation is associated with the incidence and development of cancer [24]. Our study showed that the F/B ratio of the CCA group was significantly lower than that of the HNC group, which is a good way to distinguish a healthy state from a diseased state. Considering that patients with cancer exhibit intestinal dysbiosis, we wanted to determine whether an altered F/B ratio might be associated with the serum tumor marker CA19-9 in CCA patients. We observed a lower F/B ratio in CCA patients, and the F/B ratio decreased with increasing CA19-9 levels. But the F/B ratio in the CCN group was lower than that in the HNC group, but there was no statistical significance. Hence, the diagnostic potential of the microbiome for CCA remains to be revealed.

Although the F/B ratio can distinguish healthy and disease conditions, it cannot distinguish healthy conditions and CA19-9-negative CCA patients well. Therefore, it is necessary to identify a new index to better distinguish the healthy and diseased state. In our study, we found that the B/K ratio in the CCA group was significantly lower than that in the HNC group and gradually decreased with increasing CA19-9 levels. Surprisingly, compared with the HNC group, CA199-negative CCA patients had a significantly lower B/K ratio, which could clearly distinguish healthy individuals from CA199-negative CCA patients, compensating for the deficiency of the F/B ratio.

Our study showed that CA199 was positively correlated with the NLR, PLR, and CRP but negatively correlated with the LMR and AAPR in CCA patients, so CA19-9 was correlated with markers of the systemic inflammatory response. High NLR, PLR, and CRP and low LMR and AARP were associated with advanced clinicopathological features and poor prognosis [5–7], which can be concluded that high levels of CA19-9 are associated with poor prognosis of CCA. At the same time, it was found in our study that CA19-9 was negatively correlated with the B/K ratio. This evidence implicates inflammatory-microbiota interactions in CCA. In conclusion, both the microbial inflammatory response and the systemic inflammatory response are correlated with CA19-9, and the interaction between inflammation and microorganisms plays an important role in the development of CCA, which suggests that the diagnostic potential of the microbiome combined with the serum tumor marker CA19-9 for CCA remains to be revealed.

The CONUT score system is a system for scoring immune nutritional status that was developed in 2005. A growing body of evidence has suggested that patients with high CONUT scores generally have poor nutritional and protumor immunity statuses, potentially leading to tumor invasion and metastasis [47, 48]. Patients with health problems such as obesity and cachexia exhibit several gut microbiota alterations. The CONUT score can be used to assess cachexia in patients with cancer, which may influence changes in the intestinal microbiota. Our study found that the CONUT score of the CCP group was lower than that of the CCN group, and the beneficial bacteria in the intestinal flora of the CCP group decreased, while the pathogenic bacteria increased, suggesting that CCA patients with high CA19-9 may have poor nutritional status and more serious intestinal flora disorder, which predicted a poor prognosis.

A study showed that the primary sclerosing cholangitis–inflammatory bowel disease (PSC–IBD) association may be related to the enterohepatic circulation of gut-derived molecules and possibly facilitated by increased intestinal permeability [9]. To date, few studies have been performed on CCA bile microbes, and bile and fecal microbes are affected by enterohepatic circulation. Our study revealed differences in alpha and beta diversity between the bile and fecal microbiomes as well as similarities and significant differences in species composition and relative abundances. In the future, we will further study how bile and fecal microbes interact with each other through enterohepatic circulation in CCA patients. Not only do our observations here add to the growing literature on microbiome associated with CCA, but also importantly, this is one of the pioneer clinical studies to evaluate bile microbiome in CCA, with a focus on tumor marker CA19-9. Prior studies have mostly evaluated gut microbiota in relationship to CCA and other cancers.

The present study also had certain limitations. First, we divided the patients into three groups according to CA19-9 levels to obtain the above results. With the increase in CA19-9 levels, both the microbial level index and clinical index showed corresponding changes. In the future, the sample size will be expanded to explore the optimal boundary between the CA19-9 level and clinical parameters and microbiome parameters. Second, there were fewer CCA patients with CA19-9-negative, and the current study cohort lacked HNC and CA199-negative CCA bile samples, which will be corrected in the future to clarify the bile microbiome characteristics of different cohorts. It is difficult to study human ductal bile and almost impossible to obtain samples either in a sterile way or without prior perioperative antibiotic prophylaxis. Selecting proper controls is challenging since ERC is a method associated with health risks for the examined individual and thus cannot be performed on healthy volunteers and CA19-9-negative CCA patients who do not require ERC procedures or have obstructive jaundice for ethical reasons. The obtained results were achieved based on a limited sample size, and future validation in larger cohorts may reduce the number of false-positive results and unreliability during early diagnosis of CCA. Finally, the verification of the B/K ratio and ROC curve by an external database cannot be better matched due to the lack of clinical parameters from the external database. To address these limitations, our future plans include expanding the sample size, including a higher proportion of CA19-9-negative CCA patients, and examining the potential auxiliary role of the B/K ratio in early diagnosis.

## Conclusions

This is the first study showing that the B/K ratio may serve as an effective way to distinguish healthy individuals from CA19-9-negative CCA patients and establish a microbe-based prediction model to improve the early diagnosis of CCA. However, the derived results stem from a limited sample size, emphasizing the need for extensive validation using larger cohorts across multiple centers in future studies.

### Supplementary information

ESM 1 (XLSX 13 kb)

ESM 2 (XLSX 496 kb)

ESM 3 (DOCX 24 kb)

ESM 4 (XLSX 17 kb)

ESM 5 (DOCX 643 kb)

## Data Availability

The raw sequence data presented in the study are publicly available. The raw sequence data have been deposited in NCBI Sequence Read Archive under BioProject accession PRJNA1004431.
