# Supplementary material for: A Novel Bifidobacterium/Klebsiella Ratio in Characterization Analysis of the Gut and Bile Microbiota of CCA Patients
Source: Microb Ecol. 2023 Nov 30;87:5. doi: 10.1007/s00248-023-02318-3 (PMC10687116; doi:10.1007/s00248-023-02318-3)
Supplement: Supplementary file 1 [file 248_2023_2318_MOESM3_ESM.docx]

Table S3. The demographic and clinicopathological characteristics and nutrional characteristics of the CCAs patients and the healthy normal group.

|  | CCN | CPL | CPH | HNC |
| --- | --- | --- | --- | --- |
|  | n=8 | n=17 | n=17 | n=16 |
| Gender（Male） | 5（62.5%） | 10（58.8%） | 9（52.9%） | 6(37.5%) |
| Age, years | 66.5 ± 2.299 | 62.29 ± 2.911 | 62.88 ± 2.564 | 53±8.017 |
| HBP | 5（62.5%） | 12（70.6%） | 6（35.3%） | 3（18.75%） |
| DM | 1（12.5%） | 3（17.6%） | 6（35.3%） | 1（16.7%） |
| HGB（g/L） | 142.3 ± 6.475 | 120.3 ± 3.729** | 122.4 ± 5.118* | - |
| TBIL(μmol/L) | 44.06 ± 25.8 | 148.1 ± 34.95 | 195.6 ± 30.67** | - |
| DBIL(μmol/L) | 34.91 ± 23.35 | 128.8 ± 30.73 | 170.8 ± 26.27** | - |
| NEUT(*109/L) | 3.956 ± 0.5836 | 4.022 ± 0.3465 | 5.012 ± 0.6688 | - |
| LYMPH(*109/L) | 1.925 ± 0.2234 | 1.278 ± 0.1135** | 1.252 ± 0.08679** | - |
| MONO(*109/L) | 0.505 ± 0.03601 | 0.5081 ± 0.03745 | 0.5029 ± 0.03556 | - |
| PLT(*109/L) | 211.6 ± 21.84 | 255.1 ± 20.82 | 228.4 ± 17.94 | - |
| ALB(g/L) | 42.28 ± 1.645 | 39.44 ± 0.9982 | 37.14 ± 1.674 | - |
| ALP(U/L) | 191.3 ± 23.86 | 351 ± 70.68 | 540.8 ± 95.47*# | - |
| NLR | 2.221 ± 0.3474 | 3.435 ± 0.3691* | 4.121 ± 0.5685* | - |
| LMR | 3.965 ± 0.5837 | 2.664 ± 0.263* | 2.444 ± 0.2703* | - |
| PLR | 115.9 ± 11.43 | 222.3 ± 32.88* | 201.8 ± 23.3* | - |
| AARP | 0.2761 ± 0.04726 | 0.1704 ± 0.02594* | 0.09924 ± 0.01929***# | - |
| CRP | 1.213 ± 0.5267 | 10.18 ± 2.378 | 30.18 ± 8.351 | - |

*: compared to CCN group, *P < 0.05, **P < 0.01, ***P < 0.001. #:compared to CPL group,#P<0.05.
