# Supplementary material for: A Novel Bifidobacterium/Klebsiella Ratio in Characterization Analysis of the Gut and Bile Microbiota of CCA Patients
Source: Microb Ecol. 2023 Nov 30;87:5. doi: 10.1007/s00248-023-02318-3 (PMC10687116; doi:10.1007/s00248-023-02318-3)
Supplement: Supplementary file 2 [file 248_2023_2318_MOESM5_ESM.docx]

Supplementary Figure 1

Histogram of the relative abundances in the microbiomes of the HNC and CCA groups at the genus level.

Supplementary Figure 2

Histogram of the relative abundances in the microbiomes of the HNC and CCA groups at the species level.

Supplementary Figure 3

Histogram of the relative abundances in the microbiomes of the CCN and CCP groups at the genus level.

Supplementary Figure 4

Histogram of the relative abundances in the microbiomes of the CCN and CCP groups at the species level.

Supplementary Figure 5

Histogram of the relative abundances in the microbiomes of the HNC and CCN groups at the genus level.

Supplementary Figure 6

Histogram of the relative abundances in the microbiomes of the HNC and CCN groups at the species level.
